# Isotretinoin treatment upregulates the expression of p53 in the skin and sebaceous glands of patients with acne vulgaris

**DOI:** 10.1007/s00403-022-02508-y

**Published:** 2022-12-31

**Authors:** Naglaa Fathi Agamia, Khalid Fawzi El Mulla, Naglaa Mohamed Alsayed, Rasha Mohamed Ghazala, Rania Elsayed Abdel El Maksoud, Iman Mohamed Abdelmeniem, Iman Mamdouh Talaat, Inass Ibrahim Zaki, Rana Mohamed Sabah, Bodo Clemens Melnik

**Affiliations:** 1 Department of Dermatology, Andrology and Venereology, Faculty of Medicine, University of Alexandria, Alexandria, Egypt; 2 Department of Medical Biochemistry, Faculty of Medicine, University of Alexandria, Alexandria, Egypt; 3 Department of Pathology, Faculty of Medicine, University of Alexandria, Alexandria, Egypt; 4 Department of Clinical Sciences, College of Medicine, University of Sharjah, Sharjah, UAE; 5 Department of Dermatology, Environmental Medicine and Health Theory, University of Osnabrück, 49076 Osnabrück, Germany

**Keywords:** Acne, Gene expression, Isotretinoin, p53, Sebaceous gland

## Abstract

The transcriptomic regulation induced by isotretinoin (*13-cis* retinoic acid) is still a matter of debate as short-term exposures of immortalized sebocytes with isotretinoin produced conflicting results. Based on translational evidence, it has been hypothesized that oral isotretinoin treatment upregulates the expression of the transcription factor p53. Twenty-five patients suffering from acne vulgaris were treated with isotretinoin (0.6 mg/kg body weight) for 6 weeks. Biopsies from back skin were taken before and after isotretinoin treatment for the determination of p53 expression by immunohistochemical staining, quantification of p53 protein concentration by enzyme-linked immunosorbent assay and *TP53* gene expression by quantitative reverse transcription real time PCR. Fifteen socio-demographically cross-matched healthy volunteers served as controls. Isotretinoin treatment significantly increased the nuclear expression of p53 in sebaceous glands of treated patients compared to pre-treatment levels and p53 levels of untreated controls. Furthermore, the p53 protein and gene expression significantly increased in the skin after treatment. The magnitude of p53 expression showed an inverse correlation to acne severity score and body mass index. Under clinical conditions, isotretinoin induced the expression of p53, which controls multiple transcription factors involved in the pathogenesis of acne vulgaris including FoxO1, androgen receptor and critical genes involved in the induction of autophagy and apoptosis. Increased p53-FoxO1 signalling enhanced by systemic isotretinoin treatment explains the underlying transcriptomic changes causing sebum suppression but also the adverse effects associated with systemic isotretinoin therapy.

## Introduction

Acne vulgaris is a chronic inflammatory cutaneous disorder with a complex multifactorial pathogenesis depending on increased and modified sebum production, altered upper pilosebaceous duct keratinization, loss of follicular microbial diversity with aberrant biofilm-producing phylotype colonization of *Cutibacterium acnes* (*C. acnes*), and follicular as well as perifollicular inflammation [1–6].

Sebum is the secretory product of sebocytes derived from sebaceous gland holocrine secretion [6]. Exaggerated sebocyte activity stimulated by increased insulin-like growth factor 1 (IGF-1)/IGF1 receptor (IGF1R)/phosphatidylinositol-3 kinase (PI3K)/AKT [7] and androgen/androgen receptor (AR) signalling [8] enhances and modifies sebum production exhibiting higher amounts of monounsaturated pro-inflammatory fatty acids [9, 10].

On the transcriptional level, sebaceous glands of acne patients exhibit decreased nuclear expression of the transcription factors FoxO1 and FoxO3a [11–13] and increased activity of mechanistic target of rapamycin complex 1 (mTORC1) [13–15], a key regulator of sebocyte proliferation, lipogenesis, autophagy end endocrine responses in acne pathogenesis [16–18]. Of note, FoxO1 operates as a nuclear co-suppressor of AR [19]. FoxO1 and FoxO3a are extruded from the nucleus into the cytoplasm by insulin/IGF-1/AKT-mediated FoxO phosphorylation [19, 20]

Importantly, the transcription factor p53, known as the guardian of the genome [21, 22], is critically involved in the expression of FoxO1 [23], FoxO3a [24, 25], tumour necrosis factor-related apoptosis-inducing ligand (TRAIL) [26], tumour necrosis factor receptor superfamily member 10B (TNFRSF10B; death receptor 5) [23], repression of AR [27] and IGF1R [28], and suppression of IGF-1-AKT-mTORC1 signalling [28–31], thus linking crucial transcriptional and nutrigenomic regulators involved in acne pathogenesis.

Increased mTORC1 activity promotes cell growth and anabolism [32]. Increased body mass index (BMI) has been positively associated with acne risk and severity in several studies [33–36]. Notably, p53 has been recognized as crucial player in nutrient sensing pathways and functions as a negative regulator of mTORC1 [29–31] and adipogenesis [37].

Among the various agents used for the treatment of acne vulgaris, isotretinoin (*13-cis* retinoic acid) is the most effective sebum-suppressive drug reducing skin surface and comedonal lipids [28, 38]. Isotretinoin is considered the first choice for the treatment of cystic acne [39, 40].

Translational evidence suggests that isotretinoin's desired anti-acne effects and its adverse effects including teratogenicity are based on isotretinoin-mediated apoptosis [41, 42]. In fact, Nelson et al. [43, 44] demonstrated in several studies that isotretinoin induces apoptosis and cell cycle arrest in human SEB-1 sebocytes [43] and increases the expression of the apoptotic protein TRAIL [44], which mediates the apoptotic effects of isotretinoin in human sebaceous glands.

Sebocytes are able to isomerize *13-cis* retinoic acid to *all-trans* retinoic acid (ATRA) [45]. In sebocytes, isotretinoin increases the expression of cellular retinoid acid-binding protein-2 (CRABP-2) [46], which transports ARTA into the nucleus to retinoic acid receptors (RARs) regulating gene expression [47–49]. The *CRABP2* gene promoter contains a TATA-box that is rapidly activated by ATRA through a retinoic acid response element (RARE) [48]. Compared to epidermis, CRABP-2 is strongly expressed in suprabasal sebocytes in isotretinoin-treated patients, promoting a preferential transport of ATRA to RARs in sebocytes [44, 50]; ATRA binding to nuclear RARs enhances the expression key transcription factors involved in apoptosis including forkhead box transcription factors FoxO1 and FoxO3a and TRAIL [51]. It has been demonstrated by Agamia et al. [53] that nuclear levels of FoxO1 and FoxO3a increased in sebaceous glands of patients with acne vulgaris after treatment with oral isotretinoin. It has been shown in epidermal keratinocytes that the expression of p53 is upregulated by ATRA exposure [54, 55]. Shi et al. [56] observed in human primary keratinocytes enhanced expression of p53, FoxO1 and p21 after isotretinoin exposure. As recently hypothesized by Melnik [57], isotretinoin-induced overexpression of p53 may also be the underlying pharmacological mode of action for sebocyte apoptosis and isotretinoin-mediated teratogenicity (neural crest cell apoptosis) [41, 42]. In fact, both isotretinoin and ATRA induce the expression of p53 and apoptosis in melanoma cells [58, 59]. Isotretinoin/ATRA-mediated upregulation of p53 in isotretinoin-treated acne patients may also be the underlying mechanism enhancing the expression of pro-apoptotic effectors including the p53-responsive genes *FOXO1* [23], *FOXO3A* [23, 25] and *TNFSF10* [26] promoting sebocyte apoptosis [26, 50, 54, 57]. Indeed, increased expression of ATRA-induced CRABP-2 and TRAIL have been demonstrated in the basal and suprabasal layers of sebaceous glands and skin during isotretinoin treatment of acne patients [44, 46, 60], where increased isotretinoin-mediated apoptosis activity has been observed [44].

The aim of this study was to assess the expression of p53 in the skin and sebaceous glands of acne patients before and during oral isotretinoin treatment to understand isotretinoin’s transcriptomic mode of action in the treatment of acne under clinical in vivo conditions.

## Patients and methods

### Patients and patient samples

This study was conducted on 25 patients suffering from acne vulgaris and 15 socio-demographically cross-matched healthy volunteers who served as controls. All participants were recruited from the Dermatology Outpatient Clinic of the Alexandria Main University Hospital. Approval by ethical committee as well as written informed consent was obtained from all patients and controls. All procedures were in accordance with the ethical standards of the institutional and/or national research committee and the 1964 Declaration of Helsinki.

Patients presenting acne vulgaris within the age range of 17–25 years of both sexes were included. The exclusion criteria included female patients on antiandrogen therapy or with signs of hyperandrogenism (polycystic ovaries, androgenic alopecia, hirsutism), patients with a history of prior systemic retinoid intake or antibiotic therapy during the last 6 months, patients with diabetes mellitus or other endocrine diseases. Patients were not advised to change their usual dietary habits during the study. Patients were subjected to a full history, general medical examination, and dermatological examination.

Acne severity in patients was classified using a simple acne grading system [61, 62] based on the predominant lesion and the number and locations of acne lesions. It classifies acne into four grades as follows: Grade 1: comedones and occasional papules; grade 2: papules, comedones and a few pustules; grade 3: predominant pustules, nodules and abscesses; and grade 4: mainly nodules, abscesses and widespread scarring.

All patients were given oral systemic isotretinoin for treatment of acne with a dose of 0.6 mg/kg body weight/day for 6 weeks, after full routine investigations before treatment.

### Skin biopsy

The procedure was explained to all patients. One 5 mm punch biopsy (for the immunohistochemical study) and two 2.5 mm punch biopsies (for ELISA and PCR) were taken from lesional skin on the back of the patient before isotretinoin treatment and another three biopsies were taken from residual non-scarred lesions on the back after 6 weeks of treatment.

The control subjects were those undergoing surgical procedure on the back recruited from the plastic surgery department. Single 5 mm punch biopsy and two 2.5 mm punch biopsies were taken from normal skin of the back.

### Histopathological examination and immunohistochemical detection of p53

Skin biopsies were fixed in 10% formalin. Then tissue sections were dehydrated in a series of ascending grades of ethyl alcohol (70%, 95%, 100%). Biopsy specimens were cleared in xylene then embedded in paraffin wax, sectioned by microtome and stained with Hematoxylin and Eosin stain with addition of cover slips. Histopathological examination was performed using a light microscope; all specimens were prepared for immunohistochemical staining using mouse anti-human monoclonal p53 antibody (isotype: IgG2b). (Anti-p53 antibody) [pAb122] (ab90363) (Abcam, Cambridge, U.K.) [63].

The overall staining intensities in sebaceous gland areas of the slides stained with p53 monoclonal antibodies were scored using digital image analysis with a computer-assisted light microscope. The image of each slide was captured using a 400 × objective lens. Images were viewed and recorded using an Olympus microscope (Olympus, Centre Valley, PA, U.S.A.) equipped with a Spot digital camera (Spot Imaging Solutions, Sterling Heights, MI, U.S.A.) and MATLAB software (MathWorks, Natick, MA, U.S.A.). The mean values of each reaction were based on the mean pixel number. The integrity of the colour intensity was based on grey-level transition probabilities in digitized images from dark to light. The overall intensity of staining of slides stained with p53 monoclonal antibody was scored according to nuclear expression into 0 if staining intensity is < 10%, + 1 if staining intensity is 10% ≤ 30%, + 2 if staining intensity is 31% ≤ 50% and + 3 if staining intensity is > 50% [53].

### Determination of p53 protein concentration by enzyme-linked immunosorbent assay

Skin biopsies were collected and preserved at – 80 ℃. After determination of sample weight and addition of PBS pH 7.4, samples were homogenized by hand or grinders and finally centrifuged for 3 min at a speed of 10,000 r.p.m. to remove the supernatant.

The ELISA kit (Abcam, Human p53 ELISA Kit (ab171571) was used for the determination of p53 protein. This assay is based on the principle of double-antibody sandwich technique to detect human p53 tumour protein. For further technical details, see procedure published by the manufacturer. Antibodies labelled with enzyme were added for an incubation time of 60 min at 37 ℃. After washing the plates and addition of Chromogen solution A, B, optical density (OD) values were measured for the calculation of p53 protein concentrations of the samples [64].

### Determination of *P53 gene* expression by quantitative reverse transcription real time PCR

Total RNA was extracted from 10 mg skin tissue after lysis and homogenization, using silicate gel technique provided by the RNeasy Mini Kit (Qiagen) [65]. The concentration and purity of RNA were measured at 260, 280 and 230 nm using Nano Drop 2000c spectrophotometer (Thermo Scientific, USA). A ratio of *A*_260_/*A*_280_ = 1.8–2.1 and *A*_260_/*A*_230_ = 1.8–2.1 indicates highly pure RNA. Total RNA was reverse transcribed into cDNA using high-capacity reverse transcriptase kit (Applied Biosystems™, USA, catalog no. 4368814). To detect *TP53* in tissue samples, primers had been matched to the mRNA sequences of the target genes (NCBI Blast software). GADPH was used as housekeeping gene [66].

#### P53

- 5’-AGA GTC TAT AGG CCC ACC CC-3’ (forward)
- 5’-GCT CGA CGC TAG GAT CTG AC-3’ (reverse)

#### GAPDH

- 5’-CAT GGG GAA GGT GAA GGT CGG A-3’ (forward)
- 5’-TTG GCTCCC CCC TGC AAA TGA G-3’ (reverse)

The PCR amplification was performed in a 25 µl reaction volume including SYBR green PCR Master Mix (Applied Biosystems) using ABI 7900 sequence detector (Applied Biosystems). The reaction was performed with 10 min of initial stage to activate the DNA polymerase, followed by 40 cycles at 95 °C for 15 s and 60 °C for 1 min. Single product formation was confirmed by melting point analysis, and comparative CT method was used to calculate relative gene expression with GADPH as an endogenous control. For statistical analysis of the CT values, 2-^ΔΔCT^ method was applied for each specific primer and real-time PCR [66].

### Statistical analysis

Data were fed to the computer and analysed using IBM SPSS software package version 20.0. (Armonk, NY: IBM Corp.). Shapiro–Wilk test was used to verify the normality of distribution of variables; comparisons between groups for categorical variables were assessed using *χ*^2^ test (Monte Carlo). Marginal homogeneity test was used to analyse the significance between the different stages. Mann–Whitney test was applied to compare between two groups for not normally distributed quantitative variables. Wilcoxon signed ranks test assessed for comparison between two periods for not normally distributed quantitative variables. ANOVA was used for comparing different categories. Kruskal–Wallis test was used to compare different categories for abnormally distributed quantitative variables. Pearson coefficient was used to correlate between two normally distributed quantitative variables. Significance of the obtained results was judged at the 5% level [67, 68].

## Results

### Patient data

This study was conducted on 40 subjects. Twenty-five patients suffering from acne vulgaris (18 males and 7 females) and 15 acne-free subjects served as controls (12 males and 3 females). The mean age of patients and controls was 20.08 ± 2.91 and 21.87 ± 3.07 years, respectively. There was a statistically significant difference between BMI in cases and controls exhibiting a mean BMI of 25.87 ± 2.47 kg/m^2^ in patients and 23.26 ± 3.44 kg/m^2^ in controls, respectively (*p* = 0.008*).

Clinically, there was statistically improvement in acne severity after treatment among the patients (*p* < 0.001*). Before treatment, 12 cases (48%) were grade IV, 9 cases (36%) were grade III, and 4 cases (16%) were grade II, respectively. While after treatment, 17 patients (68%) were grade I; 8 patients (32%) were grade II. Acne severity was not correlated to the age of patients while it was significantly correlated to BMI of patients before treatment.

### Laboratory findings

**Fig. 1:**
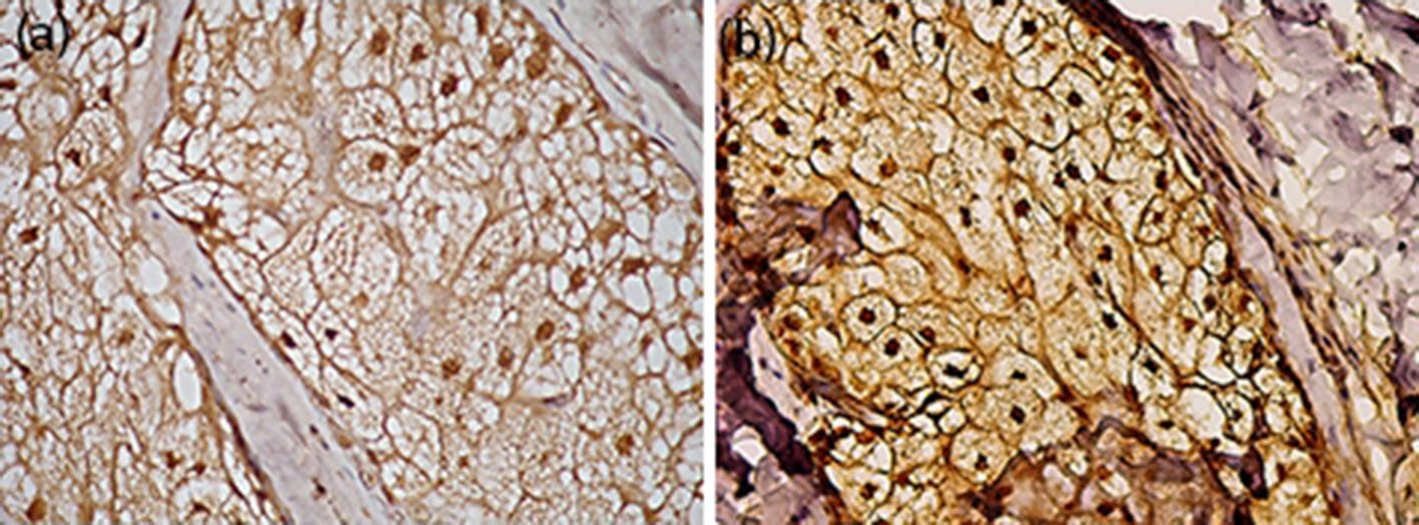
Immune staining of nuclear p53 before and after isotretinoin treatment. **a** p53 nuclear immune staining in sebaceous gland of acne patient before isotretinoin therapy. **b** Intensified nuclear p53 immune staining after 6 weeks of isotretinoin therapy

In skin biopsies taken from acne patients staining intensity of p53 increased after isotretinoin treatment compared to pre-treatment and acne-free controls. Figure 1 presents the representative immunostaining pattern with p53 antibody before and after isotretinoin treatment showing increased nuclear stain intensity in the patients’ sebaceous glands after isotretinoin treatment.

Before treatment the stain intensity of p53 was zero in 19 patients (76%), + 1 in four patients (16%) and + 2 in two patients (8%), respectively. While after treatment, the stain intensity was + 1 in five patients (20%), + 2 in eight patients (32%), and + 3 in twelve patients (48%), respectively. The difference in immunohistochemical expression of p53 before and after treatment was significant (^MH^p_1_ < 0.001*). p53 expression in the control group was significantly higher compared to the pre-treatment acne patients, (^MC^p_2_ = 0.521), while it was significantly lower when compared to the post-treatment biopsies (^MC^p_3_ < 0.001*) (Table 1).

**Table 1:** Comparison between p53 expression of patients and controls

	Cases (*n* = 25)		Control (*n* = 15)
	Before	After	
P53 protein concentration by ELISA (concentration of P53/ mg protein)			
Median (Min.–Max.)	94.8 (49.9–121)	183.3 (129.3–503)	161 (114–202)
Mean ± SD	90.78 ± 23.58	225.9 ± 105.3	160.93 ± 31.03
Significance	^Z^p_1_ < 0.001*, ^U^p_2_ < 0.001*, ^U^p_3_ = 0.052		
P53 gene expression by RT-PCR			
Median (Min.–Max.)	0.04 (0.0–0.20)	0.71 (0.48–0.98)	0.27 (0.20–0.31)
Mean ± SD	0.07 ± 0.06	0.72 ± 0.17	0.26 ± 0.04
Significance	^Z^p_1_ < 0.001*, ^U^p_2_ < 0.001*, ^U^p_3_ < 0.001*		
Immunohistochemical expression of P53			
Grade 0	19 (76.0%)	0 (0.0%)	10 (66.7%)
Grade + 1	4 (16.0%)	5 (20.0%)	2 (13.3%)
Grade + 2	2 (8.0%)	8 (32.0%)	3 (20.0%)
Grade + 3	0 (0.0%)	12 (48.0%)	0 (0.0%)
Significance	^MH^p_1_ < 0.001*, ^MC^p_2_ = 0.521*, ^MC^p_3_ < 0.001*		

p_1_: *p* value for comparing between *before* and *after*; p_2_: *p* value for comparing between *cases* (*before*) and *control*; p_3_: *p* value for comparing between *cases* (*after*) and *control*

*SD* standard deviation, *U* Mann–Whitney test, *Z* Wilcoxon signed ranks test, *MH* Marginal homogeneity test, *MC* Monte Carlo (*χ*^2^ test)

*Statistically significant at *p* ≤ 0.05

**Fig. 2:**
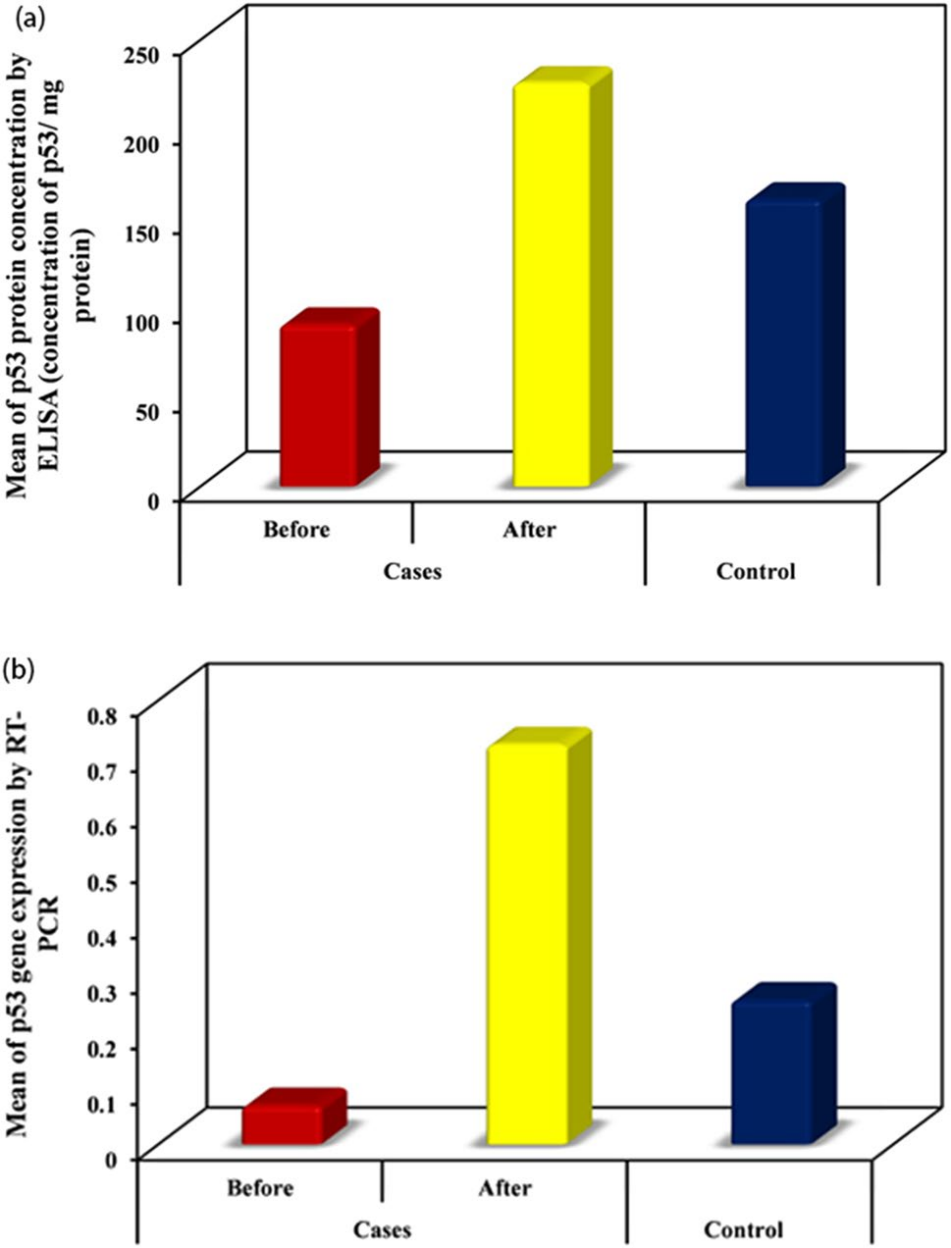
p53 protein and gene expression before and after isotretinoin therapy. **a** Illustrates the changes of p53 protein and **b** the changes of p53 gene expression of back skin biopsies of acne patients before and after 6 weeks of oral isotretinoin treatment compared to acne-free control skin biopsies

The mean of p53 protein concentration determined by ELISA before treatment was 90.78 ± 23.58 p53/mg protein. This increased significantly after isotretinoin treatment to 225.85 ± 105.34 p53/mg protein (^Z^p_1_ < 0.001*). The mean of p53 protein concentration in control subjects was 160.93 ± 31.03 p53/mg protein) (^U^p_2_ < 0.001*). Similarly, the mean p53 cDNA expression by PCR before treatment was 0.07 ± 0.06 that increased significantly after treatment to be 0.72 ± 0.17 (^Z^p_1_ < 0.001*). cDNA expression before treatment with isotretinoin was significantly lower than in controls (0.26 ± 0.04) (^U^p_2_ < 0.001*). After-treatment results were significantly higher than p53 baseline expression of controls. (^U^p_3_ < 0.001) (Table 1, Fig. 2).

**Fig. 3:**
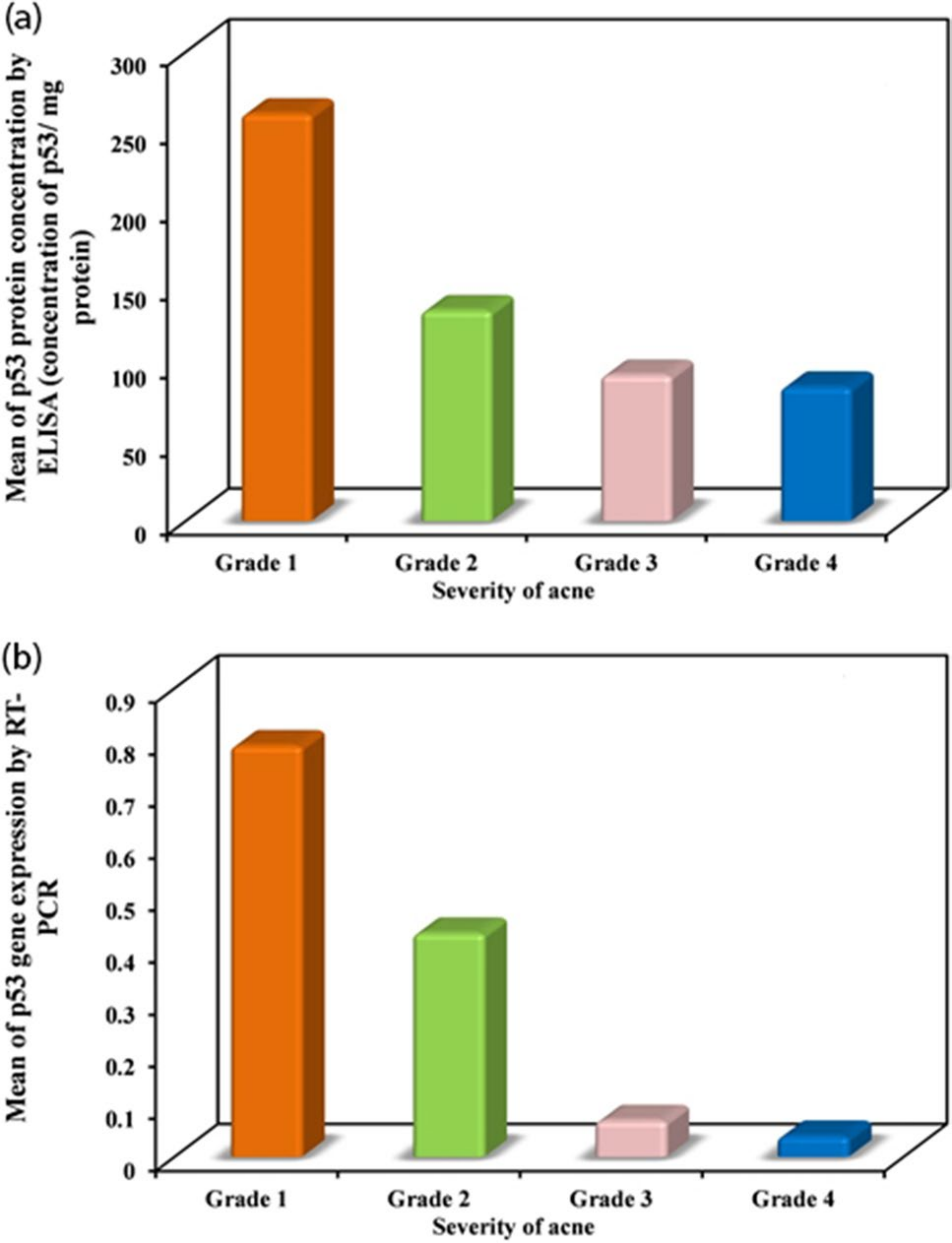
Correlation between p53 protein and gene expression with acne severity grade. **a** Shows a negative correlation between p53 protein expression and **b** an inverse relation between p53 gene expression with acne severity grade

**Fig. 4:**
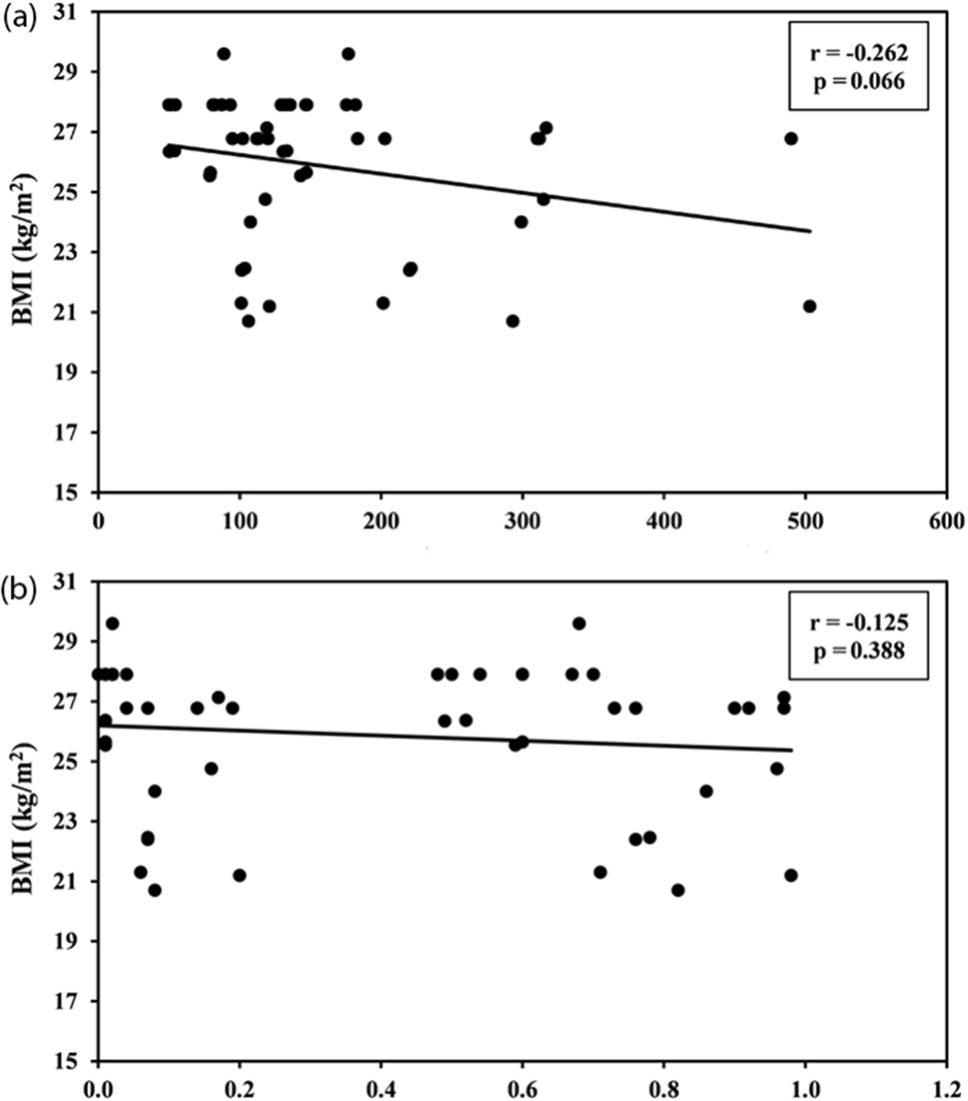
**a** Correlation between BMI and protein expression by ELISA in cases group (*n* = 50) **b** correlation between BMI and gene expression of p53 by RT-PCR in cases group (*n* = 50)

Furthermore, we could observe a negative correlation between p53 protein and gene expression with acne severity grade (Fig. 3). In addition, a negative correlation has been found between patients’ pre-treatment p53 expression and BMI (Fig. 4).

## Discussion

The sebum-suppressive effect of isotretinoin has been related to sebocyte apoptosis [41–44] with isotretinoin-mediated p21-induced cell cycle arrest [43] and upregulation of pro-apoptotic transcription factors including FoxO1 [53] and FoxO3a [53] as well as the apoptosis effector TRAIL [44, 60]. The transcription factor p53, known as the guardian of the genome [69], is a key regulator of cell fate decisions including cycle control and induction of apoptosis depending on the magnitude of p53 transcription and activation. Notably, p53 promotes the expression of the cell cycle inhibitor p21 (*CDKN1A*) [71] and the pro-apoptotic proteins FoxO1 [23], FoxO3a [23, 25], and TRAIL [26] and inhibits anti-apoptotic pro-survival effectors such as IGF1R [28], AR [27] and survivin (*BIRC5*) [72], all known p53 target genes involved in acne pathogenesis. It has been demonstrated in primary human keratinocytes and melanocytes that isotretinoin and ATRA increase the expression of p53 [54–59]. According to a recent hypothesis, isotretinoin’s mode of action and its adverse effects are related to enhanced expression of p53 [57, 72]. In fact, our study provides first experimental evidence that isotretinoin significantly upregulates the expression of p53 in the skin and sebaceous glands of acne patients after 6 weeks of oral isotretinoin therapy with the commonly used daily dose of 0.6 mg/kg body weight.

Remarkably, the skin of acne patients compared to acne-free controls exhibits lower levels of p53 expression (Fig. 2), whereas after isotretinoin-treatment p53 levels significantly exceeded p53 levels in healthy skin, pointing to a strong induction of p53 by systemic and prolonged isotretinoin exposure.

It is noteworthy to mention that p53 expression is regulated by endocrine and nutrient signalling. Increased insulin and IGF-1 signalling, which both activate the kinase AKT, result in phosphorylation and activation of E3 ubiquitin ligase mouse double minute 2 (MDM2) promoting the proteasomal degradation of p53 [73, 74]. Western diet with increased insulin/IGF-1/AKT signalling [11] may thus reduce the expression of p53, the key negative regulator of mTORC1 [29–31], which exhibits increased activity in the skin and sebaceous glands of acne patients [13–17]. The fundamental ability of mTORC1 promoting cell growth and anabolism [32] may also explain the potential relation between BMI and acne risk [33–36]. In fact, p53 is not only a tumour suppressor but has been appreciated as a crucial player in nutrient sensing pathways serving as a negative regulator of mTORC1 [29–31] and adipogenesis [37].

Importantly, activated mTORC1 is a key suppressor of autophagy [75]. Remarkably, p53 not only induces apoptosis but also stimulates autophagy [76, 77]. It has recently been shown in immortalized SZ95 sebocytes that isotretinoin treatment, partly via activation of FoxO1, increased the expression of ATG5 and induced autophagy resulting in reduced sebaceous lipid accumulation [78]. Notably, p53 can activate the expression of a large set of target genes that are involved in the autophagic programme including ATG5 [79, 80]. Autophagy is required for robust p53-dependent apoptosis. Thus, autophagy and apoptosis are two closely related p53-dependent cellular responses [81, 82]. It is thus conceivable that isotretinoin induces both p53-mediated autophagy as well as p53-induced apoptosis depending on the dose and duration of isotretinoin exposure and the resulting magnitude of p53 expression.

Moreover, it should be kept in mind that p53 is partially inactivated by simian virus 40 large T antigen in immortalized SZ95 and SEB-1 sebocytes [83, 84], which may thus not be suitable cell lines for studying p53-dependent effects of sebaceous gland regulation leading to paradoxical even acnegenic effects [85] disputed earlier [86].

There is compelling translational evidence that isotretinoin-mediated upregulation of p53 expression explains isotretinoin’s teratogenicity via p53-mediated neural crest cell apoptosis [41, 42]. Isotretinoin also induces apoptosis in primary human keratinocytes [56], melanoma cells [58, 59], rat ovarian granulosa cells [87, 88], hepatoma cells [89], associated with decreased expression of the apoptosis inhibitor survivin [89]. Increased serum levels of survivin have been reported in acne patients compared to controls [90]. Of note, survivin (*BIRC5*) expression is negatively regulated by p53 [91]. In accordance with these findings and our results, we conclude that isotretinoin-induced expression of p53 not only promotes sebocyte apoptosis in human sebaceous glands as the predominant sebum-suppressive effect but is also responsible for all isotretinoin’s adverse effects.

The most common mucocutaneous side effects of isotretinoin therapy, dry skin, have been related to increased expression of keratinocyte aquaporin 3 (*AQP3*), which damages the skin barrier and enhances transepidermal water loss causing skin dryness [92]. Notably, *AQP3* is a p53 target gene [93]. Other members of the aquaporin family, AQP1 and AQP4 [94, 95] have been linked to intracranial hypertension (*pseudotumor cerebri*), a potential adverse effect of isotretinoin [96], and appear as well to be related to upregulated p53 [97, 98]. In addition, isotretinoin-induced hypertriglyceridemia [99] is associated with increased plasma levels of apolipoprotein B100 in very low-density lipoprotein (VLDL) and low density lipoproteins (LDL) [100]. The gene encoding apoB100 (*APOB*) has been identified as p53 target gene [101].

It is important to remember that retinoids induce primary and secondary transcriptional responses depending on dose and duration of retinoid exposure [51]. Sufficient nuclear transport of ATRA via CRABP2 is mandatory for ATRA-induced transcriptomic changes [51] including isotretinoin/ATRA-induced transcriptional modification resulting in sufficient sebum suppression [46]. Increased expression of CRABP2 in isotretinoin-treated sebaceous glands of patients with acne has been observed after weeks of oral isotretinoin exposure [46], whereas short-term (6 h, 24 h) isotretinoin exposure of immortalized p53-inactivated SZ95 sebocytes did neither exhibit increased CRABP2 nor upregulated p53 or FoxO1 expression [102]. This is in contrast to our in vivo findings under clinical conditions observed in patients treated with isotretinoin for 6 weeks, whose sebaceous glands are not p53-inactivated by SV40 viral transfection [53].

Notably, p53 maintains baseline expression of common tumour suppressor genes including FoxO1 [23]. Over the last 10 years, decreased FoxO1 expression has been linked to acne pathogenesis [11, 16, 53, 103], whereas isotretinoin treatment increases FoxO1 expression in sebaceous glands of acne patients [53, 104, 105]. The p53 target gene FoxO1 is a nuclear co-suppressor of multiple transcription factors critically involved in acne pathogenesis such as AR [19], SREBF1 [106], PPARA [107] and is a crucial promoter of genes involved in apoptosis. Recent evidence indicates that FoxO1 is involved in the induction of autophagy in isotretinoin-treated SZ95 sebocytes [78]. Furthermore, FoxO1 promotes the expression of GATA6, a critical transcription factor maintaining appropriate keratinocyte proliferation and differentiation of the infundibulum of the human sebaceous follicle [108], which is deficiently expressed in sebaceous follicles of acne patients linking p53-FoxO1-GATA6 deficiency to comedogenesis.

Reduced baseline expression of the tumour suppressor p53 in acne patients compared to acne-free controls may also explain the increased risk of acne patients for common p53-related malignancies such as prostate cancer [109, 110], and breast cancer [111]. Notably, there is no observed acne and very low cancer incidence in IGF-1-deficient patients with Laron syndrome [112], who exhibit higher p53-FoxO1 signalling [113]. In contrast, Western diet with high glycaemic load and milk/dairy consumption increases insulin/IGF-1 signalling promoting AKT/MDM2-mediated proteasomal degradation of p53 [50, 114, 116], whereas forced upregulation of p53-FoxO1 signalling may contribute to the tumour suppressing effect of isotretinoin in neuroblastoma [117] and retinoid chemoprevention of non-melanoma skin cancer [118].

Taken together, our study provides experimental evidence for increased nuclear expression of p53 in sebaceous glands and skin of acne patients after oral isotretinoin treatment and substantiates that enforced p53-FoxO signalling causes all desired and adverse effects of systemic isotretinoin therapy.

## Data Availability

The data that support the findings of this study are available from the corresponding author upon reasonable request.
